# What Do Evaluation Instruments Tell Us About the Quality of Complementary Medicine Information on the Internet?

**DOI:** 10.2196/jmir.961

**Published:** 2008-01-22

**Authors:** Matthew Breckons, Ray Jones, Jenny Morris, Janet Richardson

**Affiliations:** ^1^School of Nursing and Community StudiesUniversity of PlymouthPlymouthUK

**Keywords:** Consumer Health Informatics, Internet, quality of information, complementary medicine

## Abstract

**Background:**

Developers of health information websites aimed at consumers need methods to assess whether their website is of “high quality.” Due to the nature of complementary medicine, website information is diverse and may be of poor quality. Various methods have been used to assess the quality of websites, the two main approaches being (1) to compare the content against some gold standard, and (2) to rate various aspects of the site using an assessment tool.

**Objective:**

We aimed to review available evaluation instruments to assess their performance when used by a researcher to evaluate websites containing information on complementary medicine and breast cancer. In particular, we wanted to see if instruments used the same criteria, agreed on the ranking of websites, were easy to use by a researcher, and if use of a single tool was sufficient to assess website quality.

**Methods:**

Bibliographic databases, search engines, and citation searches were used to identify evaluation instruments. Instruments were included that enabled users with no subject knowledge to make an objective assessment of a website containing health information. The elements of each instrument were compared to nine main criteria defined by a previous study. Google was used to search for complementary medicine and breast cancer sites. The first six results and a purposive six from different origins (charities, sponsored, commercial) were chosen. Each website was assessed using each tool, and the percentage of criteria successfully met was recorded. The ranking of the websites by each tool was compared. The use of the instruments by others was estimated by citation analysis and Google searching.

**Results:**

A total of 39 instruments were identified, 12 of which met the inclusion criteria; the instruments contained between 4 and 43 questions. When applied to 12 websites, there was agreement of the rank order of the sites with 10 of the instruments. Instruments varied in the range of criteria they assessed and in their ease of use.

**Conclusions:**

Comparing the content of websites against a gold standard is time consuming and only feasible for very specific advice. Evaluation instruments offer gateway providers a method to assess websites. The checklist approach has face validity when results are compared to the actual content of “good” and “bad” websites. Although instruments differed in the range of items assessed, there was fair agreement between most available instruments. Some were easier to use than others, but these were not necessarily the instruments most widely used to date. Combining some of the better features of instruments to provide fewer, easy-to-use methods would be beneficial to gateway providers.

## Introduction

While the ever-expanding source of health information might be seen as a positive step in consumer empowerment (between 36% and 55% of Internet users access online health information [1-4]), several studies have highlighted problems with the quality of the information [5-8]. Searching for relevant and reputable complementary medicine information is particularly challenging [9], in part due to methodological challenges. Schmidt and Ernst [10] report that claims are made that can put consumers at risk; in some cases, adherence to advice obtained from the Internet has had serious consequences [8,11]. This is of particular concern for people who may be vulnerable, such as those affected by cancer [12]. In the case of hydrazine sulfate poisoning [8], inaccurate and exaggerated claims of effectiveness and lack of information on side effects were blamed for misleading a consumer who assumed the substance was safe.

A 2002 study estimated that 5 million adults in England lack basic literacy [13]. Furthermore, understanding health information may be a complex process requiring more than basic literacy skills as even well-educated people can have difficulties making sense of it [14]. A US study of over 350 health sciences students showed that while many rated themselves as possessing good research skills, only a small proportion were able to demonstrate that they could identify reliable information [15]. While consumers may regularly make judgments of the quality of information received through traditional media such as newspapers, books, or leaflets, quality indicators for Internet content may not be as evident to users [16].

Consumers looking for health information are likely to select the first few links that appear on search engines and tend not to look for information about site authors or disclaimers that sites may make [17]. Studies have found that when consumers evaluate the quality of health information on the Internet, they tend to rely onendorsement by government agencies or professional organizations, their own perception of reliability of the website source, and the understandability of the information [18,19].

Several strategies have been designed to help health information seekers access high-quality information, including codes of conduct, gateway sites (portals), and evaluation instruments. The development of instruments has received the greatest attention, and some suggest that their use by consumers can educate the user as to the characteristics of a good quality website [16], but the behavior of the majority of consumers would suggest that gateways may be the best approach. Evaluation instruments can still provide a method for researchers to help choose links for gateways. Evaluation instruments work on the premise that they can identify “quality” sites on the assumption that sites that conform to indicators of quality are likely to contain accurate information. Accurate information is defined as being based on a gold standard of information in the field. While it is not possible for someone with no domain knowledge to assess accuracy, it may be that instruments can be used to help make judgments on quality and hence predict accuracy.

Gateways are collections of sites that have been prescreened and deemed of high enough quality to be approved by a governing organization. Examples of these are Healthfinder [20] and Intute [21]. Although maintaining portals can be labor intensive, organizations providing services such as complementary medicine for cancer need to be able to recommend sites to their patients. There are many instruments that have been designed, ranging from simple checklists to long and complex documents providing detailed accounts of assessment methodologies, and organizations running a portal need to choose which to use. Three characteristics that would seem important are (1) agreement with other instruments when rating a website, (2) ease of use, and (3) longevity. On the latter, many instruments seem to have a very limited life span. In 1998, for example, Jadad and Gagliardi identified 47 instruments used to rate the quality of health information on the Internet [22], but 4 years later [23], only six of these instruments still existed.

Our study was conducted in a center providing complementary care for people affected by cancer. The aim of this study was to identify website evaluation instruments and to assess their performance when used by a researcher to evaluate a sample of 12 websites on complementary medicine for people with breast cancer. In particular, we asked the following:

- Do the instruments use the same criteria, and do they agree on the ranking of websites?
- How easy are the different instruments for a researcher to use?
- Are these instruments likely to remain in use such that future readers will appreciate the assessment method used?
- Could we identify a pragmatic approach to identify good quality complementary medicine websites using existing instruments?

## Methods

### Literature Search for Evaluation Instruments

We defined an evaluation instrument as something that an Internet user could use to assess the quality of a website containing health information. To identify evaluation instruments, search terms were based on previous papers that had attempted to identify instruments for evaluating the quality of Internet health information [22,24,25]. The databases Medline, AMED, BNI, CINAHL, EMBASE, and PsychInfo were searched in February 2007 using the following terms: “evaluat* OR assess* OR rating OR rat* OR ranking OR rank* OR quality OR criteria AND website* OR world wide web OR Internet.” This achieved results of 29,622, 233, 123, 14,859, 8678, and 10,593, respectively. When in excess of 1000, the most recent results from each database were examined.

In addition to the above databases, the search engines Google, MSN, Yahoo, and WebCrawler were searched using the following terms: “evaluate OR assess OR rating OR criteria OR quality AND websites OR Internet.” This achieved results of 212,000,000, 25,410,704, 38,400,000, and 28, respectively. With the exception of WebCrawler (28 results), the first 100 results of each were examined. Relevant research papers and bibliographies were also examined for relevant references. Several large studies that had attempted to systematically search for and identify instruments for assessing Internet health information were found [22-25].

Instruments were selected if they provided the user with explicit instructions for evaluating the quality of a website containing health information. While the HON Code of Conduct (HONcode) has been mentioned as a gateway site, its evaluation criteria were included in this report.

### Internet Search for Complementary Medicine Websites

To search for websites to be assessed, a search for “complementary (medicine OR therapies) AND breast cancer” was performed in February 2007 using the Google search engine. This resulted in 1,170,000 hits. The first six results were selected on the basis that people are most likely to look at only the first few results produced by a search engine [17]. Another six results were chosen purposively to obtain a selection of sites with different purposes and origins: sites belonging to charities, sponsored sites, and sites selling products.

### Assessment of Websites

The 12 websites were evaluated using each of the 12 evaluation instruments (ie, 144 assessments). Each site was given a mark using the individual scoring system for each instrument, which was then converted to a percentage score. Some instruments gave negative scores for failing to meet criteria; therefore, it was possible for a negative score to be obtained. Sites were then ranked from 1 (best) to 12 (worst) based on these scores.

### Comparison of Evaluation Instruments

The range of criteria used by the identified instruments was compared to the nine main criteria identified by the Health Improvement Institute and Consumer Reports WebWatch (HIICRW) [26] in 22 health information rating instruments. Agreement between instruments was assessed by a correlation matrix using Spearman rank correlation on the instruments’ ranking of the websites.

### Illustrative Comparison of Best and Worst Sites

The range of content on each site made comparison against a gold standard impossible. Nevertheless, we sought some “face validity” in that sites ranked as “good” or “poor” using these evaluation instruments matched with common sense. Statements made on the site ranked the best by the sum of the 12 instruments were compared to those on the site ranked the worst.

### Citation Search for Use of Evaluation Instruments

A citation search on Web of Science was carried out using the original papers describing the instruments. A sample of papers that cited the original paper was reviewed, and an estimate was made of the number of papers that had used the tool. A citation search on Google using the instrument’s http address was carried out. A sample of websites that cited the original Web address of the tool was checked to see if the citation was correct. The number of citations on Web of Science or Google was classified as low (less than 10), medium (11-100), or high (greater than 100).

### Longevity of Instruments

The URLs of instruments that had been identified in four previous studies were checked (as part of the literature search) to see if they still existed. Instruments were reported as unavailable if the original URL was not found and searching the original site or Google for the instrument did not locate it.

## Results

### Evaluation Instruments Available

A total of 39 instruments that disclosed their criteria and aimed to help users identify good quality information online were identified. Of these, 12 met our inclusion criteria (Table 1); the other 27 were excluded (Table 2). Instruments were selected if they provided the user with a set of objectives and closed questions that could be applied to a website containing health information by someone with no prior subject knowledge and without having to look at sources other than the website being assessed. Reasons for exclusion of the 27 instruments included the following:

- A consumer could not apply the instrument without further knowledge (eg, “Is the information written by reputable authors?”).
- Scoring details were unavailable (eg, Instructions stated to score each criterion on a scale of 1-5, but no further information was given as to how to allocate a value.).
- Questions were not objective (eg, “Are the graphics attractive?”).
- Instrument was not designed specifically for health information.
- Questions were open ended (eg, “What are the author’s qualifications?”).
- Instrument took the role of a tutorial that gave tips on how to find reliable health information on the Internet but was not applicable as an instrument.

### Websites Sampled

Table 3 shows the websites that were rated using the instruments; four of the sites were run by UK charities, two sites were selling products, and three sites were US sites offering cancer treatment. One site was run by a network of health professionals, one site was funded by advertising on its site, and one site was funded by sponsors.

**Table 1:** Evaluation instruments used in the study

Evaluation Instrument	Method of Assessment	Ease of Use (Researcher Assessment)	Comprehensiveness (HIICRW Criteria Met, out of 9)
WEB FEET HEALTH Collection: Criteria for Site Selection (WEB FEET) [27]	24 statements: agree or disagree	+ Straightforward questions − Time consuming to apply	7
HONcode [28]	8 desirable properties	+ Short tool, quick to apply + Each element includes guidelines	4
Emory University Rollins School of Public Health, Health-Related Web Site Evaluation Form (Emory) [29]	36 statements: +1 disagree, +2 agree, 0 N/A Score: 0-60	+ Interpretation of score − Time consuming	7
University of Michigan Web Site Evaluation Checklist (Michigan) [30]	43 questions, with variety of positive and negative scores Score: −80 to +80	+ Printable rating form + Interpretation of score − Time consuming − Complex scoring system	6
Kellogg Library (University of Dalhousie), Evaluation of Health Information on the Internet (Kellogg) [31]	31 questions: agree or disagree	+ Simple questions, straightforward to use − Time consuming	8
DISCERN Quality Criteria for Consumer Health Information (DISCERN) [32]	16 questions on 5-point analogue scale from “No” to “Yes” Overall score: 1-5	+ Explanation of criteria + Interpretation of score − Answers on visual analogue scale more difficult / time consuming	5
National Center for Complementary and Alternative Medicine (NCCAM), 10 Things to Know About Evaluating Medical Resources on the Web (NCCAM) [33]	10 questions each with explanation	+ Clear guidance of how to use criteria − No scoring system	6
US Pharmacist tool (Pharm) [34]	15 questions with yes/no answers	+ Easy to apply − No scoring system	6
Minervation Validation Instrument for Health Care Web Sites (Minervation) [35]	Semi-automated tool requires URL of site being assessed Drop-down menus to answer questions of content and usability Rating automatically calculated Score: 0-100%	+ Automated usability check + Drop-down menus, fast + Interpretation of score	5
Nicoll LH, author’s guidelines (Nicoll) [36]	Mnemonic (PLEASED) with yes/no questions, each with author justification of importance	+ Quick to use + Explanation of criteria − No scoring system	5
Silberg et al, authors’ guidelines (Silberg) [37]	4 items that should be met	+ Quick to apply − Does not assess aspects unique to Internet information	3
Sandvik score (Sandvik) [38]	7 questions, each with 3 options scored 0-2 Score: 0-14	+ Quick to apply + Simple scoring system	3

**Table 2:** Evaluation instruments excluded

Evaluation Instrument	Requires Further Knowledge	Scoring Details Unavailable	Questions not Objective	Not Health Specific	Open-Ended Questions	Tutorial
Quality Criteria for Health Related Websites [39]	x					
Net Scoring Criteria to Assess the Quality of Health Internet Information [40]		x				
Criteria for Evaluating the Quality of Health Information on the Internet [41]	x		x			
Administration Design Quality Web Site Evaluation Method [42]	x					
Evaluating Websites [43]			x	x		
Navigating the Health Care System: How to Evaluate Health Information on the Internet [44]					x	x
Rating Criteria and Excellence Awards [45]			x			
Clean Bill of Health Award [46]				x		
Health Website Rating (HWR) Project: HII Health Website Rating Instrument (HWRI) [47]					x	
Clearing House^*^		x				
Best of the Web in Mental Health: Rating Guidelines [48]			x			
Commentary: Measuring Quality and Impact of the World Wide Web [49]						x
Evaluating Internet Health Information: A Tutorial From the National Library of Medicine [50]						x
MedlinePlus Guide to Healthy Web Surfing [51]						x
Taking Charge of Health Information [52]					x	
How to Evaluate Health Information on the Internet: Questions and Answers [53]					x	
How to Find the Most Trustworthy Health Information on the Internet [54]			x			
Internet Detective [55]			x			x
Internet for Health and Well-Being [56]			x			x
Suggestions for Using the Internet to Find New Cancer Treatments [57]						x
Internet Health Coalition^*^						x
How to Judge the Quality of a Web Site [58]			x			x
Intute: Health and Life Sciences Evaluation Guidelines [59]			x			x
Best Practice Web Assessments: Evaluation Criteria [60]		x		x		
Evaluation Form Used for LASIK Websites [61]			x			
Quality Standards for Medical Publishing on the Web [62]				x		
Evaluating Internet Resources in Complementary and Alternative Medicine [63]				x		x

^*^Instruments became unavailable between initial search (February 2007) and final submission of paper (November 2007).

**Table 3:** Twelve websites on complementary medicine and breast cancer

Website	Purpose of Site	Reason for Inclusion
Breast Cancer Care [64]	A UK charity aimed at providing information and support for people affected by breast cancer. National Health Service (NHS) information partner.	1st on Google
Breast Cancer Haven [65]	A UK charity that runs day centers offering support, information, and complementary therapies to people affected by breast cancer.	2nd on Google
CancerHelp UK [66]	A UK information service for people with cancer and their families run by the Cancer Research UK charity for cancer and cancer care.	3rd on Google
Imaginis [67]	An independent resource for information and news on breast cancer and related women’s health topics.	4th on Google
MD Anderson Cancer Center [68]	An information service run by the University of Texas MD Anderson Cancer Center that offers medical services to people with cancer.	5th on Google
Cancer Treatment Centers of America [69]	An information service run by Cancer Treatment Centers of America, a network of cancer treatment hospitals and facilities offering conventional and complementary therapies.	6th on Google
Cancerbackup [70]	A cancer information charity offering information, practical advice, and support for cancer patients, their families, and caregivers.	Charity
Heart Spring [71]	A resource for alternative and complementary health information funded by advertising and product sales.	Sponsored: product advertisements
Issels Treatment [72]	Information produced by Issels Medical Center, a private organization offering alternative treatment for cancer.	Private cancer center
Alternative Cancer [73]	A site run by an individual selling a guide to complementary and alternative cancer treatments.	Commercial
MedicineNet [74]	Medical information written by a network of medical professionals.	Sponsored: product advertisements
Elbee Global [75]	A site selling herbal medicines for people with cancer.	Commercial

### Assessment of Evaluation Instruments

#### Comprehensiveness

The HIICRW [26] defined nine criteria that an assessment should have. Assessment of each evaluation instrument against the HIICRW criteria showed considerable variation, implying little consensus on quality markers for websites. Although assessment of more criteria may not mean an evaluation instrument is superior, it is interesting that two of the better-known instruments (HONcode and DISCERN) assessed relatively few of the items described by HIICRW (see Table 1).

#### Ease of Use

Table 1 shows the researcher’s subjective view on the evaluation instruments’ ease of use. Time taken is an important component of ease of use; answering Michigan University’s 43 questions was extremely time consuming, in contrast to the automated Minervation instrument, which could be applied very quickly. Some instruments were not designed to provide numerical scores. It was useful to have some interpretation of how many criteria a website should meet for it to be thought of as being good or bad quality. Instruments varied in the explanation of their criteria. It was helpful to have further guidance available to answer questions, such as provided by HONcode and DISCERN.

### Ranking of Websites

Table 4 shows the percentage score for each of the 12 websites and the ranking from best (1) to worst (12) by each instrument and overall. It was notable that the well-known UK charity site Cancerbackup came only 4th in the overall ranking and that the WEB FEET tool ranked it 7th, way behind the Elbee Global website. The HONcode ranked it 5th, on par with the Elbee Global website. Overall, the best site was Imaginis and the worst, Alternative Cancer.

### Comparison of Evaluation Instruments

Table 5 shows the agreement (rank correlations) among instruments on the ranking of the 12 websites from best to worst. Where there is a significant correlation (eg, between Michigan and Kellogg), using either tool would give similar results. This showed that WEB FEET and HONcode seemed to assess different characteristics than the other instruments.

### Recognition and Use of Instruments

Recognition, citation, and use of instruments are necessary if they are to survive. Table 6 shows the Web of Science level of citation by other papers describing the instruments and the citations of the instruments’ website addresses on Google.

### Comparison of Best and Worst Sites

Table 7 shows illustrative extracts of statements made in the best and worst ranked sites. As would be expected, the best site (Imaginis) took a balanced and cautious approach to all claims. The Alternative Cancer site, rated the worst, made claims that were exaggerated or difficult to prove or disprove.

### Longevity of Evaluation Instruments

Table 8 shows four studies that previously searched for and identified evaluation instruments and how many of those instruments were still available in November 2007.

**Table 4:** Ranking and percentage score of websites

Evaluation Instrument	Breast Cancer Care	Breast Cancer Haven	Cancer Help	Imaginis	MD Anderson	Cancer Treatment Centers	Cancerbackup	Heart Spring	Issels	Alternative Cancer	MedicineNet	Elbee Global
WEB FEET	6 75%	8 67%	1 92%	2 83%	4 79%	10 58%	7 71%	2 83%	10 58%	12 46%	9 63%	4 79%
HONcode	9 50%	2 88%	9 50%	2 88%	1 100%	5 63%	5 63%	2 88%	9 50%	9 50%	5 63%	5 63%
Emory	6 89%	7 86%	2 95%	3 92%	3 92%	8 84%	1 97%	8 84%	11 63%	11 63%	3 92%	10 68%
Michigan	7 28%	6 45%	2 50%	1 53%	2 50%	8 21%	5 48%	9 16%	10 14%	11 1%	4 49%	12 −14%
Kellogg	5 70%	5 70%	1 90%	1 90%	3 77%	9 50%	4 73%	5 70%	10 33%	11 27%	8 63%	11 27%
DISCERN	8 66%	4 76%	2 80%	5 74%	2 80%	9 52%	1 89%	5 74%	10 46%	12 31%	7 69%	11 39%
NCCAM	5 60%	5 60%	3 70%	1 90%	1 90%	10 50%	3 70%	5 60%	5 60%	11 40%	5 60%	12 20%
Pharm	6 80%	7 73%	2 93%	2 93%	4 87%	9 60%	1 100%	8 67%	12 33%	10 47%	4 87%	10 47%
Minervation	4 73%	7 62%	2 79%	3 74%	6 71%	5 64%	1 82%	11 46%	9 60%	10 48%	7 62%	12 34%
Nicoll	3 71%	7 57%	1 86%	3 71%	3 71%	9 29%	1 86%	8 43%	12 14%	9 29%	3 71%	9 29%
Silberg	7 25%	7 25%	3 75%	3 75%	1 100%	7 25%	3 75%	1 100%	7 25%	7 25%	3 75%	12 0%
Sandvik	7 64%	7 64%	2 79%	2 79%	1 86%	10 29%	6 71%	2 79%	9 36%	11 21%	2 79%	12 7%
**Overall rank**	**8**	**7**	**2**	**1**	**3**	**9**	**4**	**6**	**10**	**12**	**5**	**11**

**Table 5:** Spearman nonparametric correlation coefficients between evaluation instruments, based on assessment of the websites

	WEB FEET	HONcode	Emory	Michigan	Kellogg	DISCERN	NCCAM	Pharm	Minervation	Nicoll	Silberg	Sandvik
WEB FEET	1.00											
HONcode	.35	1.00										
Emory	.51	.25	1.00									
Michigan	.48	.38	.87^*^	1.00								
Kellogg	.71^*^	.39	.84^*^	.89^*^	1.00							
DISCERN	.55	.47	.86^*^	.77^*^	.87^*^	1.00						
NCCAM	.55	.39	.78^*^	.89^*^	.92^*^	.82^*^	1.00					
Pharm	.53	.28	.98^*^	.88^*^	.87^*^	.84^*^	.80^*^	1.00				
Minervation	.30	−.02	.85^*^	.79^*^	.79^*^	.70	.75^*^	.85^*^	1.00			
Nicoll	.55	.14	.97^*^	.82^*^	.83^*^	.82^*^	.74^*^	.97^*^	.82^*^	1.00		
Silberg	.51	.51	.61^†^	.66^†^	.71^†^	.71^*^	.75^*^	.64^†^	.37	.59^†^	1.00	
Sandvik	.59^†^	.51	.72^*^	.83^*^	.82^*^	.77^*^	.85^*^	.73^*^	.48	.70^†^	.93^*^	1.00

^*^Correlation is significant at the 0.01 level (2-tailed).

^†^Correlation is significant at the 0.05 level (2-tailed.

**Table 6:** Number of citations in Web of Science and Google (classified as low, medium and high) suggesting use of instruments (NPI: no paper identified)

Evaluation Instrument	Web of Science	Google
WEB FEET	NPI	Low
HONcode	Med	High
Emory	NPI	Medium
Michigan	NPI	Low
Kellogg	NPI	Low
DISCERN	Med	High
NCCAM	NPI	High
Pharm	NPI	Low
Minervation	NPI	Low
Nicoll	Not cited	Low
Silberg	High	Low
Sandvik	Med	Med

**Table 7:** Comparison of statements from websites rated best and worst

Best Site (Imaginis)	Worst Site (Alternative Cancer)
“...anecdotal evidence reveals that many alternative or complementary medicines may be beneficial to patients, extensive research is still needed to determine whether non-traditional medicines are truly effective.”	“Proven Therapies. Includes a list of successful, long-standing alternative treatments from around the world going unused by the conventional medical system. There is one reason they are the oldest - in the hands of experienced practitioner they work! For example: the very successful nutritional based Gerson therapy. It has been used by untold thousands of people worldwide for over 50 years.”
“Chinese herbs have been shown to lessen the side effects of chemotherapyand acupuncture has been shown to reduce nausea (a possible side effect of chemotherapy and other drug therapies).”	“*Every day worldwide, quietly behind the scenes, there are over 100 proven alternative therapies used successfullyagainst cancer. (Get a FREE list of the 78 most popular below) The problem is, nobody bothers to tell the public. Plus, conventional cancer doctors (MD Oncologists) are not taught anything about them in medical schools. This must change!”*
“Not all alternative or complementary medicines are safe.”	“The one true secret to success: There are six basic types of proven alternative cancer treatments, and you must use them all together.”
“In a recent studypublished in the *Journal of the National Cancer Institute*, researchers found that advanced breast cancerpatients with high stress levels were less likely to live as long as patients who coped well with stress.”	“Anvirzel® A new weapon against cancer and AIDS from Ozelle Pharmaceuticals - a herbal extract which is nontoxic and causes no adverse side effects. Closed clinical trials are showing that the drug is especially effective against prostate and breast cancer. The materials of the company promoting Anvirzel. say that Dr Ozel treated 494 cancer patients with the extract, resulting in a high rate of success. The company has organized phase I and II trials in Ireland, and states that the trials confirmed the efficacy of the extract in cancer. They say the patients were improved in their quality of life as well as regression of cancer, reporting no notable side effects. Best results were said to be in prostate, lung and brain cancers. Sarcomas showed stabilization.”
“Some preliminary studies have shown that vitamins may help reduce risk of breast cancer or treat the disease.”	“Artemisinin A Chinese herb, sweet wormwood (*qinghao* in Chinese). In test tube studies, breast cancer cell research resulted in a 28% reduction of breast cancer cells treated only with artemisinin, and an amazing 98% decrease in breast cancer cells within 16 hours that were treated with artemisinin and an iron-enhancing molecule, transferrin. These treatments had no significant effect on normal human breast cells. This research pointed to the involvement of free iron in the toxic effect of artemisinin toward cancer cells, basically sparing healthy cells. (‘Selective toxicity of dihydroartemisinin and holotransferrin toward human breast cancer cells,’ Life Sciences 70 {2001) 49-56.”

**Table 8:** Instruments identified in previous studies still available in 2007

Study	Year of Study	No. of Instruments Identified	No. of Instruments Available in November 2007
Jadad and Gagliardi [22]	1998	14	3
Kim et al [25]	1999	27	7
Gagliardi and Jadad [23]	2002	5	1
Bernstam et al [24]	2005	17	3

## Discussion

### Limitations of This Study

Our study has some limitations. Selection of instruments, website ratings, and HIICRW criteria comparison were performed by only one researcher. Possible interobserver variation may mean that some instruments eligible for inclusion may have been missed and that some excluded may have been included by other reviewers. Due to the nature of the instruments being searched, they do not lend themselves to very specific search terms, meaning that our searches produced many results. Nevertheless, we may have found more tools by examining a greater number of search results or by searching other databases. Two instruments were excluded only for the reason that they were not health specific and, in retrospect, that exclusion criterion may not have been warranted.

Application of the evaluation tools to particular websites may also have produced different results with other researchers. Bernstam et al, in a recent study [76], suggested that some quality criteria may have poor interobserver reliability. However, there is likely to be more variation (both intraobserver and interobserver) in the values attributed to individual characteristics of an assessment tool. When combined to give an overall rank, as we have done in this study, tools are more likely to give consistent results.

### What This Study Offers

Although our study has limitations, our experience has a useful message for several groups of people:

- For those assessing or developing gateways who may wish to use an evaluation instrument, this study provides information that may help select an instrument.
- For authors of evaluation instruments, we identified those features that may be desirable to ensure their instrument is useable and useful.
- For information seekers, we show which properties to look for when selecting an instrument and suggest which instruments may be preferable to others.
- For developers of complementary medicine websites, we show the need to use “technical markers of quality” to ensure that their site achieves high scores when assessed by instruments.

Our study also suggests that the popular HONcode may assess quality in a different way than other instruments.

### The Quality of Websites

Developers of websites or gateways on complementary medicine need some method to check the quality of what they are presenting, and users of their websites need to be able to assess for themselves, and to believe, the claim that this is a quality website. What does quality mean? Provost et al [77] define quality as the levels of excellence which characterize the content of the site based on accepted standards of quality. At the very least, it should mean that the information presented is evidence based and the evidence is available to be checked.

### The Gold Standard Approach

Impicciatorre et al [78] were among the first to assess the reliability of Web page information by comparing it against a gold standard. Others have followed this approach [7,79,80], but in every case, they have been able to focus on specific pieces of information or advice that have an available gold standard. For example, Pandolfini et al [81] compared information on the management of cough in children against a gold standard. Assessing quality in this way is time consuming, and in cases where websites present information on a broader range of topics, not a feasible option. Having some sort of evaluation that allows a quicker test of quality is therefore an attractive option, and for this reason, numerous evaluation instruments have been devised.

### Does the Evaluation Instrument Approach Act as Good Proxy for Quality of Information?

Pandolfini et al [81] examined 19 Web pages and noted that no relationship was found between technical aspect, content completeness, and quality of information as compared to a gold standard. However, only one page received a high score on comparison against the gold standard, and this page also scored high on the other two measures. In our study, we have not assessed against a gold standard, but a simple comparison of the content of the best and worst sites using evaluation instruments shows our approach to have face validity. However, we should remain cautious. While instruments are designed to assess the quality of information, they are concerned with quality indicators and can therefore not take into account the accuracy of an individual piece of information. Eysenbach et al [5] are of the opinion that it is unlikely that a universal set of criteria could be developed that would predict the quality of health information websites as there are complex relationships between quality indicators and actual quality of information. While the results of our study suggest that websites rated higher by the evaluation instruments seem typically less likely to contain exaggerated claims, Walji et al [82] analyzed 150 websites dealing with the use of ginseng, ginkgo, and St. John’s wort and concluded that domain-independent criteria may not be appropriate for identifying complementary and alternative medicine websites, suggesting that consumers should rely on authoritative providers of information. There may be specific challenges in accessing high-quality information on complementary medicine, but there are several initiatives aimed at providing high-quality, evidence-based information, including the Cancer Specialist Library [83], National Center for Complementary and Alternative Medicine (NCCAM) [84], and Complementary and Alternative Medicine Evidence OnLine (CAMEOL) [85].

### Validity, Reliability, and Agreement of Evaluation Instruments

The majority of available instruments have not been tested for reliability [24,86] or validity [86], and few include information describing the development process [82]. DISCERN and the Minervation tool appear to be the only ones that discuss the fact that their instruments have been tested for reliability and validity. Even among researchers, there is likely to be observer variation on various criteria. Bernstam et al [76] examined the degree to which two raters could reliably assess 22 popularly cited quality criteria on a sample of 42 complementary and alternative medicine websites and found poor agreement on 8/22. Good definition of the quality criteria should improve agreement, but the level of agreement between most of the instruments used in this study shows that complete “accuracy” may not be that important. Two of the instruments, HONCode and WEB FEET, did not have good agreement with the other 10 in ranking the best to worst sites. It is not clear why this is. So although HONcode is used frequently, we felt it safer to use those instruments that agreed as most of the other instruments seemed to address most aspects identified by the HIICRW.

### Ease of Use

Five of the 12 instruments were time consuming to apply. Bernstam et al [24] took the view that any tool containing more than 10 criteria was too long for routine use and that the majority of available instruments are not user friendly. Although instruments should be comprehensive, and while it may be useful to ask a wide range of questions about a site, it is important that the application of an instrument is practical. Our study suggests that greater coverage of criteria is not necessarily achieved by asking a large number of questions, although if a tool is too short it is unlikely that it could cover a wide range of criteria. There was a great deal of variation in usability of the instruments. The Minervation tool contains an automated feature that allows entry of an URL. It produces an accessibility rating, leaving the user to select answers to questions of reliability and usability from drop-down menus. It then allocates scores for each section, an overall score, and gives a rating of the site in terms of “poor,” “fair,” or “good.” These automated features are in contrast to an instrument such as the one developed by Emory University, which was very time consuming to apply. Some instruments feature further guidance to assist the user in answering the questions, which was considered a useful attribute.

### Range of Criteria

Eight of the instruments contained criteria concerning accessibility; although differing between instruments, this element asked questions about website design, layout, and if there was a search engine included on the page or appropriate links for navigation. While accessibility might not seem directly related to the quality of the information contained in the pages, it is extremely important in terms of the usefulness of the site.

Many websites “lost marks” as they did not display information concerning authorship. Eysenbach et al feel that this may be more related to convention than quality as it is not usual for organizations to display names of individual authors, and this is not necessarily an indicator of quality [5]. The way that the instrument’s question is phrased may be crucial in informing users of the quality of a site. Concerning authorship, some would ask “Is the name of the author disclosed?” which, in itself, may show that the site has a good transparency policy, but it does not add clarity to questions of quality as it is still not known if the author is suitably qualified to write on a particular topic. Similarly, regarding currency of the information, “Does the site display the date on which it was last updated?” is not as valuable as “Has the site been updated in the last 6 months?” Hence, an instrument covering the same criteria as another may achieve a different rating due to different wording of its questions.

### Number and “Shelf Life” of Evaluation Instruments

Bernstam et al [24] apparently identified 273 instruments; however, they included tools such as “top traffic” that could not be utilized by an Internet user. They identified only seven instruments that could be applied by Internet users. We did not attempt to identify instruments that could not be applied to individual sites by an information seeker.

One problem with any technology assessment method is that if the method is no longer supported or in use, citation of the results by the gateway developer becomes obsolete. Studies [22,23] and examination of previous reviews have shown that tools previously developed are no longer in use. Our study also found that the number of instruments has been reduced. It may be that people have begun to use instruments already in existence rather than to develop new ones. We examined citation of papers and Web addresses to estimate the current popularity of instruments on the basis that more popular technologies are more likely to survive. (In another field, the story of the VHS tape outliving the apparently technically better Betamax provides an example of the importance of “being popular.”) Some of the instruments that we reviewed (eg, Kellogg), although they showed agreement with other instruments and were easy to use, may not survive because they have no critical mass of use.

### The Ultimate Evaluation Instrument

We aimed to identify the best method for assessing websites for inclusion in a gateway on complementary medicine for breast cancer. No one tool seemed to be the answer. The three most-cited instruments on Google appeared to be DISCERN, HONcode, and NCCAM. HONcode does not seem to agree with the rankings produced by other instruments and seemed to have some quirks in its rankings. DISCERN seemed more difficult to apply than NCCAM, so if we chose one tool, it would be NCCAM. (This supports Walji’s assertion that complementary medicine requires domain-specific criteria.) However, we think that the authors of instruments might benefit from merging their methods to produce one tool. This has recently been argued by Provost et al [77] in reporting the development of the WebMedQual scale. They argued that harmonization of Internet-based health information evaluative efforts would benefit all users and international researchers. They reviewed the literature on rating scales and identified 384 different items used by 26 scales. Four expert reviewers rated items, eliminated duplicates, and reworded or deleted items that were not clear, meaningful, or measurable, that were thought unimportant, too general, or vague, or that could not be feasibly ascertained by an experienced but nonmedical Internet user. They ended up with the following constructs: content (19 items), authority of source (18 items), design (19 items), accessibility and availability (6 items), links (4 items), user support (9 items), confidentiality and privacy (17 items), and e-commerce (6 items). They claimed that their scale, consisting of 8 categories, 8 subcategories, 95 items, and 3 supplemental items to assess website quality, was the first step toward a standard tool that would be easy to use. However, from our experience of using NCCAM and other instruments, we question whether an instrument requiring 98 items would be quick and easy to use.

A recently developed method of assessing websites containing health information, CLUE W (personal communication, Philippe Desjardins, Laval University, 2007), is designed to assess the clinical usefulness of information to a health professional. Interestingly, this instrument calculates the usefulness of a site from a formula that incorporates validity and relevance of the information on the site as well as the work required to use this information. This instrument has undergone an extensive development process involving many health professionals. With many instruments already in existence, it will be interesting to see how much attention this new assessment method will attract.

Another new method, FA4CT [87], published after our search, differs from the checklist approach by asking users to compare information they find with information on other sites; only if discordant information is found, a checklist (the CREDIBLE checklist) is used. This is referred to by the authors as a second generation educational model. Although this approach does not guarantee that information will be compared to a gold standard, it is claimed that this method of assessment is similar to the process that experts go through when searching for, and checking, the accuracy of information on the Internet. New methods such as FA4CT may make the checklist approach obsolete, but in the meantime, this study gives those developing gateways a practical guide as to which assessment instruments may be useful.

## Abbreviations

HIICRW: Health Improvement Institute and Consumer Reports WebWatch
HONcode: HON Code of Conduct
